# Complete plastome sequence of *Flueggea virosa* (Roxburgh ex Willdenow) Voigt (Phyllanthaceae): a medicinal plant

**DOI:** 10.1080/23802359.2020.1778554

**Published:** 2020-07-06

**Authors:** Hong-Tao Wang, Hong-Xin Wang, Zhi-Xin Zhu, Hua-Feng Wang

**Affiliations:** Hainan Key Laboratory for Sustainable Utilization of Tropical Bioresources, College of Tropical Crops, Hainan University, Haikou, China

**Keywords:** *Flueggea virosa*, plastome, phylogeny, genome structure, Phyllanthaceae

## Abstract

*Flueggea virosa* is a tropical plant of the Phyllanthaceae family, which has high medicinal value. Here, we report and characterize the complete plastome of *F. virosa.* The complete plastome is 154,961 bp in length and contains the typical structure and gene content of angiosperm plastome, including two inverted repeat (IR) regions of 27,575 bp, a large single-copy (LSC) region of 83,397 bp and a small single-copy (SSC) region of 16,414 bp. The plastome contains 130 genes, consisting of 80 unique protein-coding genes, 30 unique tRNA gene, 4 unique rRNA genes (5S rRNA, 4.5S rRNA, 23S rRNA and 16S rRNA). The overall A/T content in the plastome of *F. virosa* is 63.10%. The complete plastome sequence of *F. virosa* will provide a useful resource for the conservation genetics of this species as well as for phylogenetic studies in Phyllanthaceae.

## Introduction

*Flueggea virosa* (Roxburgh ex Willdenow) Voigt is a tropical plant in the Phyllanthaceae family which is an evergreen shrub or small tree ranging from 1 to 6 m tall (Li et al. 2008). It is produced in the provinces of East China, South China and southwest China, and in the mountain shrubs with an altitude of 100–2000 meters (Yan et al. 2019). All parts of *F. virosa* are used as medicine for eczema, rheumatoid arthritis, etc. At present, the complete plastome information and systematic position of *F. virosa* has been rarely studied and reported by other scholars. Hence, the genetic and genomic information is essential needed to aid to its resource exploitation and conservation. Here, we report and characterize the complete plastome of *F. virosa* (GenBank accession number: MT424755, this study) in an effort to benefit *F. virosa* germplasm collection, conservation and future breeding.

In this study, *F. virosa* was sampled from Baoting county in Hainan province of China (109.69° E, 18.62° N). A voucher specimen (Wang et al. GPSII-001) and its DNA was deposited in the Herbarium of the Institute of Tropical Agriculture and Forestry (code of herbarium: HUTB), Hainan University, Haikou, China.

The experiment procedure is as reported in Zhu et al. (2018). Around six Gb clean data were assembled against the plastome of *Populus lasiocarpa* (KX641589.1) (Rivarola et al. 2011) using MITO bim v1.8 (Le-Petit-Quevilly, France) (Hahn et al. 2013). The plastome was annotated using Geneious R8.0.2 (Biomatters Ltd., Auckland, New Zealand) against the plastome of *Populus lasiocarpa* (KX641589.1). The annotation was corrected with DOGMA (Wyman et al. 2004).

The plastome of *F. virosa i*s found to possess a total length 154,961 bp with the typical quadripartite structure of angiosperms, contains two Inverted Repeats (IRs) of 27,575 bp, a Large Single-Copy (LSC) region of 83,397 bp and a Small Single-Copy (SSC) region of 16,414 bp. The plastozme contains 130 genes, consisting of 80 unique protein-coding genes (seven of which are duplicated in the IR), 30 unique tRNA genes (seven of which are duplicated in the IR) and 4 unique rRNA genes (5S rRNA, 4.5S rRNA, 23S rRNA and 16S rRNA). The overall A/T content in the plastome of *F. virosa* is 63.10%, which the corresponding value of the LSC, SSC and IR region were 63.40%, 69.00% and 57.80%, respectively.

**Figure 1.**
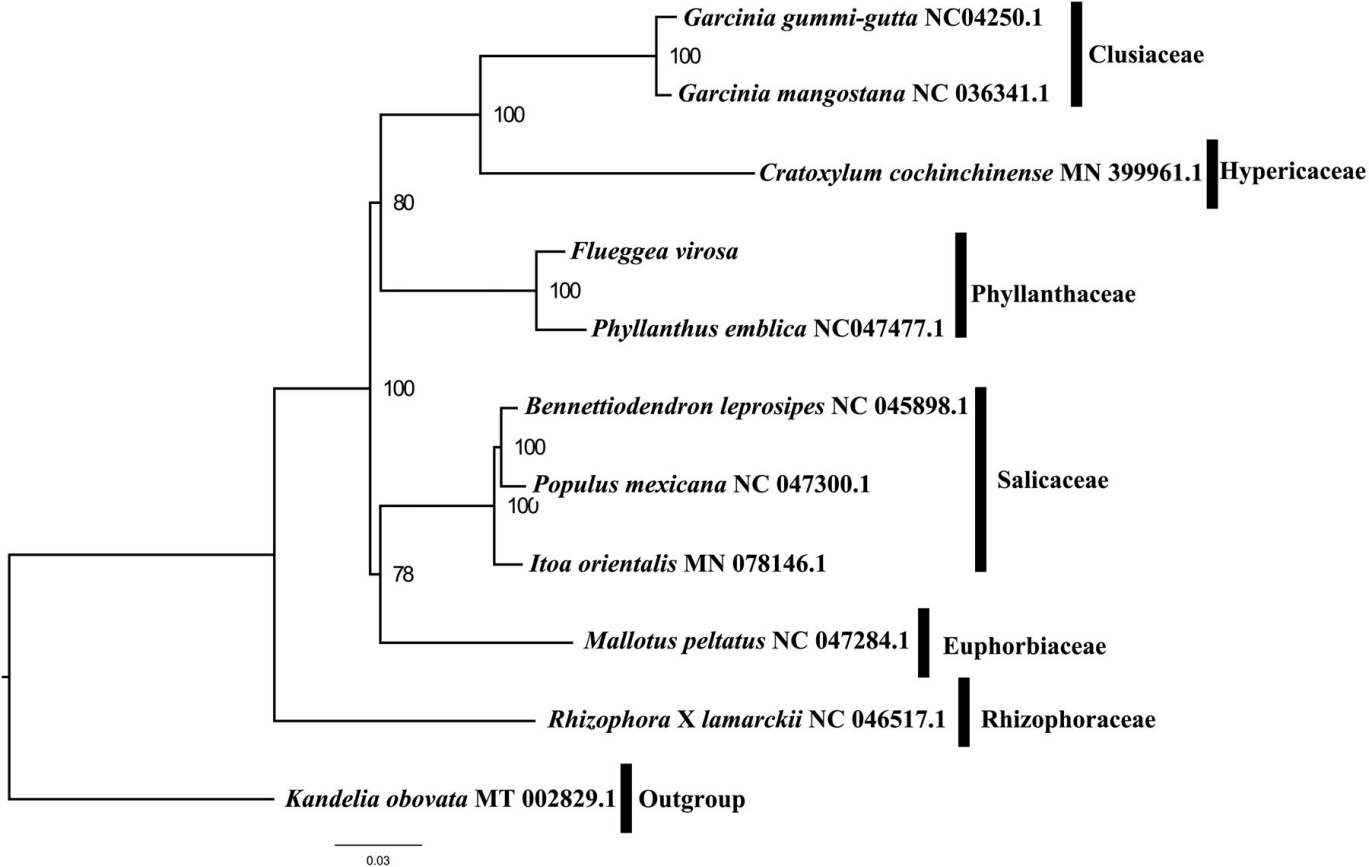
The best ML phylogeny recovered from 11 complete plastome sequences by RAxML. Accession numbers: *Flueggea virosa* (GenBank accession number, MT424755, this study), *Bennettiodendron leprosipes* NC_045898.1, *Cratoxylum cochinchinense* MN_399961.1, *Garcinia_gummi-gutta* NC_047250.1, *Garcinia mangostana* NC_036341.1, *Itoa orientalis* MN_078146.1, *Kandelia obovata* MT_002829.1, Mallotus peltatus NC_047284.1, *Phyllanthus emblica* NC_047477.1, *Populus mexicana* NC_047300.1, *Rhizophora* x *lamarckii* NC_046517.1.

We used RAxML (Stamatakis 2006) with 1,000 bootstraps under the GTRGAMMAI substitution model to reconstruct a maximum likelihood (ML) phylogeny of eleven published complete plastomes of Phyllanthaceae, using *Kandelia obovata* as outgroups. The phylogenetic analysis indicates that *F. virosa* is closer to *Phyllanthus emblica* than other species in this study (Figure 1). Most nodes in the plastome ML trees were strongly supported. With the complete plastome sequence of *F. virosa* plastome now at hand, its resource exploitation and conservation project can be better proceeded, and phylogenetic studies of Phyllanthaceae can be explored more sufficiently.

## Data Availability

The data that support the findings of this study are openly available in GenBank of NCBI at http://www.ncbi.nlm.nih.gov, reference number MT424755.
